# Mitochondrial and metabolic dysfunction in Friedreich ataxia: update on pathophysiological relevance and clinical interventions

**DOI:** 10.1042/NS20200093

**Published:** 2021-05-17

**Authors:** David R. Lynch, Garrett Farmer

**Affiliations:** Division of Neurology, Departments of Neurology and Pediatrics, The Children’s Hospital of Philadelphia, University of Pennsylvania School of Medicine, Philadelphia, PA 19104, U.S.A.

**Keywords:** biomarkers, clinical trial, mitochondrial biogenesis

## Abstract

Friedreich ataxia (FRDA) is a recessive disorder resulting from relative deficiency of the mitochondrial protein frataxin. Frataxin functions in the process of iron–sulfur (Fe–S) cluster synthesis. In this review, we update some of the processes downstream of frataxin deficiency that may mediate the pathophysiology. Based on cellular models, *in vivo* models and observations of patients, ferroptosis may play a major role in the pathogenesis of FRDA along with depletion of antioxidant reserves and abnormalities of mitochondrial biogenesis. Ongoing clinical trials with ferroptosis inhibitors and nuclear factor erythroid 2-related factor 2 (Nrf2) activators are now targeting each of the processes. In addition, better understanding of the mitochondrial events in FRDA may allow the development of improved imaging methodology for assessing the disorder. Though not technologically feasible at present, metabolic imaging approaches may provide a direct methodology to understand the mitochondrial changes occurring in FRDA and provide a methodology to monitor upcoming trials of frataxin restoration.

## Background

Friedreich Ataxia (FRDA) is a recessive disorder beginning in childhood or juvenile years that causes progressive ataxia, dysarthria, loss of sensation, and loss of coordination [1–3]. In some individuals it also leads to cardiomyopathy, scoliosis, diabetes, sensorineural hearing loss, and optic neuropathy. FRDA is caused by biallelic mutations in the *FXN* gene, which codes for the small mitochondrially targeted protein frataxin [4–6]. The most common mutation in FRDA is an expansion of a naturally occurring guanine-adenine-adenine (GAA) repeat in intron 1 (96%) that decreases but does not totally eliminate transcription of *FXN* mRNA. Approx. 4% of individuals have point mutations or deletions [7–9]. All forms of *FXN* mutations decrease the levels of functional frataxin, making FRDA primarily a disease of frataxin deficiency with all disease manifestations reflecting dysfunction downstream of frataxin loss.

Frataxin carries a mitochondrial targeting sequence, and its relative absence leads to dysfunction of multiple mitochondrial processes. The primary function of frataxin appears to be in synthesis of iron–sulfur (Fe–S) clusters in the mitochondria, and facilitating their introduction to enzymes containing this prosthetic group [10–12]. Classically, such enzymes include many of the enzymes of oxidative phosphorylation and the Krebs cycle, but many other enzymes also utilize Fe–S clusters [13]. The partial loss of frataxin in FRDA leads to deficient activity of such enzymes including aconitase, complex I and complex II [14–16], decreasing adenosine triphosphate (ATP) production and producing downstream dysfunction in mitochondrial activity and reactive oxygen species (ROS) production [17–19].

**Figure 1:**
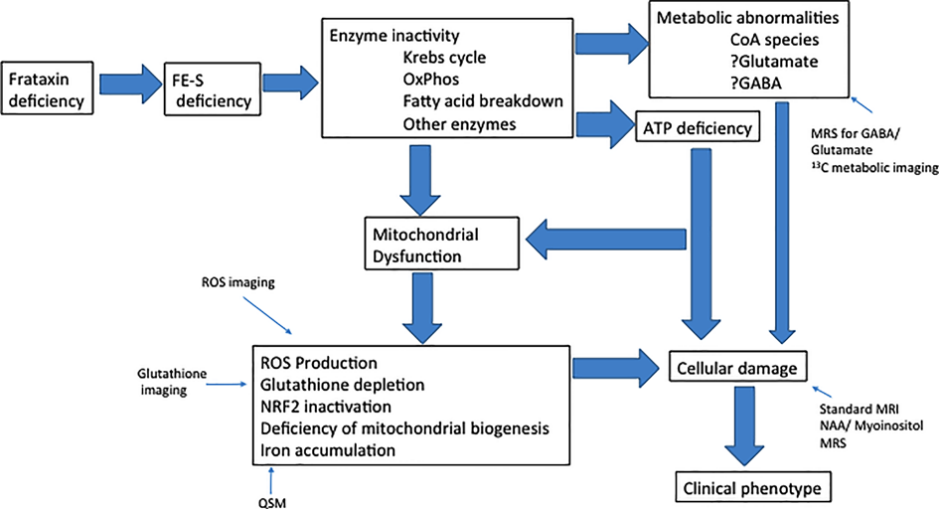
Schematic pathophysiology of FRDA Possible sites of imaging are also included.

While initial characterization of mitochondrial dysfunction in FRDA concentrated on ROS production, other specific secondary processes likely mediate large components of the FRDA pathophysiology. These include iron accumulation and ferroptosis, depletion of endogenous antioxidant systems such as those controlled by nuclear factor erythroid 2-related factor 2 (Nrf2), and deficient mitochondrial biogenesis [20–23]. Along with primary enzymatic deficiency from Fe–S cluster deficiency, these collectively alter metabolism in many systems in FRDA, including iron metabolism, glutathione synthesis, and lipid metabolism. Such mechanisms have been reviewed here previously [20] (Figure 1). In the present work, we concentrate on new variations on these topics as well as further developments linking such mechanisms to clinical therapeutics and clinical monitoring.

### Iron-mediated pathophysiology

Conceptually, FRDA is a disorder of iron distribution rather than simply overload. Iron accumulates in the mitochondria in FRDA and animal models based on frataxin deficiency, although the cytoplasm is iron deficient in FRDA, and patients systemically behave as if they are iron depleted [24–26]. In early models, mitochondrial iron accumulation was proposed as a direct contributor to ROS production through Fenton chemistry. Such a direct but simple role for iron accumulation in FRDA has largely been dismissed based on three types of observations. First, iron accumulation is not consistent across FRDA models or in brain regions in which the pathology of FRDA is most significant [27,28]. Second, direct demonstration of ROS production in patients with FRDA has proven difficult in humans [29]. Finally, direct iron chelation has not proven successful, though there is some evidence for selective effects of deferiprone in heart [30]. Deferiprone, a targeted iron chelating agent, improved cardiac hypertrophy in a modest size study but had no effects on neurological function. Overall, such results require a refinement of the role of iron in the pathophysiological mechanisms in FRDA to address the emerging concept of ferroptosis.

Ferroptosis is initiated by inhibition of the enzyme, glutathione peroxidase-4 (GPX4) in conjunction with lipid peroxidation and iron accumulation [31–34]. It is distinct from classical apoptosis, involving no nuclear involution but mitochondrial morphologic changes instead. Inducers of ferroptosis include agents that deplete glutathione, and those that inhibit or destroy GPX4. Iron accumulation is crucial in ferroptosis through promotion of lipid peroxidation. Thus, two of the known promoters of ferroptosis (iron overload, glutathione depletion) create a predisposition to this mechanism in FRDA. In fibroblasts from FRDA patients, cell death occurs in response to erastin, which initiates ferroptosis by blocking the glutamate-cysteine antiporter and thus inhibiting glutathione synthesis. Ferroptosis inhibitors block such cell death in FRDA fibroblasts whereas caspase 3 inhibitors (which block apoptosis but not ferroptosis) have no effect. Such results identify ferroptosis as a major cell death mechanism in FRDA models.

It also provides a novel direction for FRDA-related drug development. As lipid peroxidation plays a crucial role in ferroptosis, introduction of distinct membrane-destined lipids less susceptible to peroxidation could ameliorate this process. Oleic acid has been suggested to be less amenable to peroxidation, and a novel stereoselective oleic acid derivative decreases ferroptosis in FRDA fibroblasts [35]. In addition, lipophilic phenothiazine agents also have antiferroptotic activity in FRDA-derived cells, identifying a different class of agents for treatment of FRDA [36] (Table 1).

**Table 1:** Novel mitochondrially targeted agents for FRDA

Agent	Mechanism	Status	References
Oleic acid derivative	Ferroptosis inhibitor	Preclinical	[35]
Novel lipophilic phenothiazines	Ferroptosis inhibitor	Preclinical	[36]
RT-001	Ferroptosis inhibitor	Pivotal trial	[37,38]
PTC 743	Ferroptosis inhibitor	Pivotal trial	[39]
Omaveloxolone	Nrf2 activator	Pivotal trial	[50,52,53]
Letiriglitozone	PPARgamma activator	Preclinical	[68]
Elamipretide	Cardioplipin facilitator	Early trials	[69–73]

Agents discovered before the understanding of ferroptosis also may have antiferroptotic activity in FRDA. One approach is to lower the intrinsic susceptibility to lipid peroxidation by replacement of hydrogen with deuterium in lipid side chains of membrane lipids [37,38]. The commercial agent RT001, 11,11-D2-ethyl linoleate, blocks FRDA-mediated cell death in the BSO-iron model, consistent with an inhibition of ferroptosis. In a phase I/II clinical trial, the agent was well tolerated, and subjects improved peak workload during a maximal exercise. It is presently in a phase III trial with results expected in late 2021.

In addition, a second agent linked to ferroptosis, vatiquinone (α-tocotrienol quinone, EPI 743, PTC 743) has entered clinical trials in FRDA [39]. This agent inhibits 15 lipoxygenase and blocks ferroptosis in FRDA cells *in vitro*. In a moderate-size clinical trial in adults with FRDA, it failed to reach its primary endpoint in the 6-month double-blind portin. However, subjects who remained on agent for 2 years progressed less than matched individuals from natural history data. Consequently, it is presently in a phase III trial in less progressed individuals with FRDA and being evaluated for a longer duration.

### Nrf2 and endogenous antioxidant defenses

Many lines of evidence demonstrate the depletion of endogenous antioxidants in FRDA. Superoxide dismutase (SOD) levels are decreased in FRDA models and fail to induce in response to frataxin knockdown, and multiple models of FRDA demonstrate altered homeostasis of the endogenous antioxidant glutathione [40,41]. Such protective mechanisms are controlled transcriptionally by the antioxidant response element (ARE) in DNA, and the transcription factor Nrf2. Nrf2 levels are abnormal and mislocalized in FRDA cells and tissue from many different FRDA models [42–47]. Nrf2 activation increases transcription of mRNA for endogenous antioxidant enzymes such as SOD, glutathione S-transferase (GST), and NADPH: quinone oxidoreductase (NQO1), making the dysfunction of Nrf2 in FRDA a therapeutic target in FRDA. Ubiquitination of Nrf2 controls its localization and turnover; the ubiquitin E3 ligase Keap 1 provides the primary mechanism mediating these events. ROS production during mitochondrial dysfunction can modify the sulfhydryl residues of Keap I, dissociating it from Nrf2 and allowing Nrf2 to enter the nucleus and activate ARE containing genes. However, to some degree Nrf2 activation is also controlled by other E3 ligases such as Hrd1, an E3 ligase associated with ER stress [48].

Nrf2 activation with several agents improves cell viability and mitochondrial function in cells from FRDA patients. Such agents include dimethylfumarate (DMF), omavaloxolone, and at lower potency sulforaphane. Each increases Nrf2 levels by binding to Keap1, but their properties *in vivo* and *in vitro* vary to some degree. This may reflect individual pharmacokinetic features of such agents, but they also may not have identical targets of action. For example, omavaloxolone not only increases Nrf2 levels but also inhibits nuclear factor κ B (NFK-B), and DMF also raises frataxin levels both in cell culture and *in vivo*. No such effect on frataxin level has been clearly shown for omavaloxolone [49,50]. Consequently, these distinct Nrf2 activators may have varying effects in clinical trials. Such agents also may block ferroptosis, thus demonstrating the overlapping nature of pathophysiological mechanisms downstream from frataxin deficiency [51].

Clinical trials with Nrf2 activators have shown initial success [50,52–54]. Two successive studies with omavaloxolone demonstrated benefit on the neurological exam-based marker of FRDA known as the mFARS, as well as biomarkers of Nrf2 activity such as ferritin and on FRDA-based abnormal metabolism. It also provided benefit on activities of daily living in some subgroups of patients. Perhaps most importantly, there was evidence for endurance of the effects of omavaloxolone. Benefit was not immediate but accrued over 6 months with persistence to 1 year, differentiating it from previous studies of antioxidants. In clinical trials of exogenous antioxidants, apparent benefit was almost immediate and began to wane within 6 months such that no sustained benefit could be identified [55–58]. This potentially differentiates the temporal effect of transcription factor-mediated control of endogenous antioxidants from the transient effect of exogenously administered compounds. Although not yet approved for treatment of FRDA, studies of omavaloxolone provide proof-of-concept for augmentation of the endogenous antioxidant response as a target in FRDA.

The Nrf2 pathway offers other targets for intervention [48,59]. These include the alternative ubiquitin ligase HRD1 as well as the downstream enzymes such as those controlling thioredoxin and glutaredoxin. Theoretically, each of these could act synergistically in control of antioxidant defenses with Keap1-mediated control of Nrf2 degradation. Interestingly, the ubiquitin ligase Hrd1 is most commonly associated with control of ER stress, a process implicated in some models of FRDA [60]. This could provide a pathologic feedback cycle in which loss of Nrf2 leads to ER stress, which leads to further loss of Nrf2 and a potentiation of the pathophysiology of FRDA.

### Mitochondrial biogenesis

A second pathway downstream from mitochondrial dysfunction is the peroxisome proliferator-activated co-activator 1-α (PGC1α)/peroxisome proliferator-activated receptor-γ (PPARγ) pathway of mitochondrial biogenesis [61–65]. Frataxin deficiency leads to decreased levels of PGC1α in multiple models of FRDA, which, as reviewed previously, should then cause specific metabolic abnormalities and decreased mitochondrial biogenesis. Such metabolic abnormalities, including decreased levels of oxidative phosphorylation and fatty acid oxidation, and failure to renew mitochondria through their biogenesis could directly mediate the pathophysiology of FRDA [66]. Thus, this pathway represents a pharmacological target in FRDA.

Still, targeting PGC1α and its co-activator PPARγ has proven difficult. The results of a trial of pioglitazone, a PPARγ activator, have never been reported, but presumptively led to no benefit [67]. More recently, a related drug letiriglitazone has shown promise in animal and cellular models [68]. This brain penetrant, biologically active metabolite of pioglitazone, increases frataxin levels and ameliorates cell death in mouse dorsal root ganglion cell models of FRDA; it also improves altered motor function in one mouse FRDA model. Finally, it augments fatty acid B oxidation (independent of frataxin levels) and increases markers of mitochondrial biogenesis. It thus provides a possible therapeutic approach in FRDA by reversing the effects of PPARγ/PGC1α down-regulation.

The mitochondrial biogenesis pathway is not completely independent of the effects of Nrf2 as agents such as DMF have actions on both. This again suggests the possibility that multiple agents could act synergistically in augmenting downstream pathways that protect against frataxin deficiency. In addition, many agents that directly improve the mitochondrial response to frataxin deficiency (letiriglitazone, DMF) also increase frataxin levels secondarily. Another such agent is SS-31 (Elamipretide) [69–71]. This small peptide stabilizes cardiolipin in mitochondrial membranes and improves mitochondrial function. It also improves bioenergetics specifically in FRDA models and can raise frataxin levels in some situations [72–74]. This may reflect an indirect effect of cardiolipin on the level of frataxin [74].

### Extra-mitochondrial frataxin

Since the identification of the mutation in FRDA, most conceptualizations have viewed FRDA as a variant upon mitochondrial disease. However, cytosolic frataxin may also be involved. Erythrocytes make a specific form of frataxin without a mitochondrial targeting sequence, derived from a specific splice variant [75]. In mice, cytosolic forms of frataxin appear in multiple tissues, though the mechanism of their origin is not clear [76]. At present, the function of cytosolic frataxin is unknown although it could aid is cytosolic Fe–S cluster syntheses. In addition, its role in the pathogenesis of FRDA is unclear [77].

## Tracking FRDA with mitochondrial biomarkers and imaging

While mitochondrially targeted approaches may be important in therapy of FRDA, definitive therapy requires restoration of frataxin. Gene therapy, protein replacement and epigenetics-based reactivation of the *FXN* gene offer the best opportunities for sustained improvement across multiple tissues [1–3,78]. However, clinical trials of frataxin restoration require a method for confirming re-expression of frataxin in affected tissues such as heart, skeletal muscle, and brain. Most methods to measure frataxin are sufficiently invasive to be problematic in clinical trials, meaning that frataxin re-expression must be monitored by assessment of downstream processes. Imaging of mitochondrial pathophysiology *in vivo* thus becomes a crucial component of assessment of frataxin restoration.

### Magnetic resonance imaging/spectroscopy background in FRDA

Magnetic resonance imaging/spectroscopy (MRI/MRS) provides non-invasive and inexpensive techniques to image not only brain anatomy, but also metabolic function [79]. In diseases like FRDA and other ataxias, MRI can clinically provide diagnostic information by observing structural changes in specific anatomic areas and allows for monitoring of the structural changes in brain and spinal cord that correlate with neurological decline [80]. Clinical MRI studies in patients with FRDA using 3T MRI scanners can assess gray matter integrity while such scanners also create diffusion tensor images that identify previously unsuspected abnormalities in white matter in FRDA [81–88]. MRS assessing myoinositol and n-actylaspartate (NAA) can quantify the pathological changes chemically, perhaps before axons cell bodies die. However, while highly quantitative, such structural imaging is not usually needed for diagnosis or management, and its utility as a biomarker is limited as it captures mainly the fixed features of neurodegeneration. Essentially, present methods monitor the later stages in the pathophysiology of the disease and may be useful in assessing response to therapies acting on this aspect of disease but cannot readily identify the earlier steps. In contrast, chemically based imaging approaches may be needed to monitor the early, biochemical events in FRDA, before structural damage appears. Such techniques also serve as pharmacodynamic markers that may detect response to frataxin restoration more rapidly than structural imaging. Imaging approaches that capture metabolic events immediately downstream from frataxin as well as methods to image secondary processes reflecting mitochondrial dysfunction could provide imaging approaches for following the course of frataxin restoration in FRDA.

### Iron accumulation imaging techniques

Quantitative susceptibility mapping (QSM) uses MRS to quantify iron content in the brain [89], which could provide a useful biomarker in FRDA [90] through its potential link to iron accumulation [91]. Iron accumulation in the dentate nucleus correlates with clinical status and may improve in response to proposed therapy [91]. However, atrophy may precede iron accumulation, suggesting that iron accumulation likely lies relatively far downstream from the primary genetic defect. Ideally, quantifying iron accumulation would be most beneficial in measuring the events leading to ferroptosis, but the present data suggest it reflects a more empiric marker of neuronal change without direct link to the early pathophysiology. QSM thus could track components of disease progression but may not monitor the earliest events following frataxin loss or restoration.

### γ-Aminobutyric acid, glutamate, and Krebs cycle intermediates

In order to image markers closer to frataxin deficiency than accumulation of iron in FRDA, one could examine various metabolic abnormalities and enzymes altered by loss of frataxin-enhanced Fe–S cluster formation, such as Krebs cycle intermediates and compounds metabolically derived from the Krebs cycle. γ-Aminobutyric acid (GABA) and glutamate are synthesized from Krebs cycle intermediates to some degree and are key molecules in mediating metabolic dysfunction in the brain based on their role as neurotransmitters [92]. Their levels may reflect metabolic dysfunction in early FRDA based on synthetic differences, but in later disease levels of GABA and glutamate could reflect the progression of disease based on neuronal dropout. In particular, imaging these neurotransmitters could provide a method for identifying neuronal loss more selectively; for example, in FRDA, the neuronal loss in the dentate nucleus reflects loss of glutamatergic but not GABAergic cells [93]. Spectroscopic assessment GABA levels should reveal in increase over time as glutamatergic cells die. Both GABA and glutamate have been successfully imaged and quantified with 3T MRI scanners [94–96] but have not been reported in FRDA. Still, though both molecules could provide quantifiable markers of metabolic abnormalities in early disease, they are still several enzymatic steps removed from frataxin deficiency in the pathophysiology of FRDA [95–97]. Other metabolic alterations should also be proximal to cell damage or iron accumulation though such markers also may not identify the earliest features of frataxin restoration. However, even if the enzymes within the Krebs cycle could be measured, metabolism within the CNS involves complex shuttling of molecules between cells and could make exact interpretations very complex.

### Glutathione and ROS imaging and measurement

Like many small molecules in the brain, glutathione production and regeneration are impacted by mitochondrial dysfunction. Glutathione has been successfully imaged and quantified using 3T MRS techniques, though images obtained at this field strength may lack the fidelity and accuracy for detecting small changes in clinical trials [85]. Along with the 3T imaging glutathione, 7T MRS imaging can also assess glutathione as well [98]. Ultra-high MRI uses 7T scanners that are primarily used for research on human subjects, but it has not been used regularly in clinical studies. 7T images can be heavily affected by the smallest movements; in some cases, subjects’ heartbeats could move the brain by under a millimeter, altering the results [98]. Its safety in children is also unknown. While research has been conducted using 7T imaging in FRDA adults in the past [99], the clinical features of ataxia (uncontrolled movement) might confound such studies. Still this type of imaging provides significant potential in being able to quantify glutathione and can create much clearer brain images and potentially more useful biomarkers for measurement in FRDA.

Quantifying and tracking the development of ROS could provide another potential biomarker for disease progression in FRDA, looking specifically at the decline of mitochondrial function (which should lead to an increase in ROS production). ROS have been quantified and measured using fluorescent probes both *in vitro* and *in vivo* [100,101]. While this technology and advancements in research techniques could prove useful in the future, they have not been used in humans or any clinical setting. Currently, no one technique satisfies all of the constraints required to constitute an ideal imaging technique for ROS in humans; however, a combination of anatomical and functional imaging techniques may allow this technology to develop clinical utility [102]. Much like the other biomarkers, this marker is substantially downstream from frataxin deficiency. However, this information could be valuable in clinical and research settings to measure mitochondrial dysfunction related to FRDA, as well as, provide a sensitive manner for answering the question of whether ROS production actually occurs in FRDA, an ongoing pathophysiological question [29].

### ATP deficiency and imaging

ATP deficiency in FRDA results from inactivity of Fe–S containing enzymes and ultimately, frataxin deficiency. In cell culture, ATP levels provide a marker of cell viability as well, and both in cell culture and in FRDA patients ATP levels parallel disease features. Thus, the ability to measure and quantify ATP and the rate at which it changes in FRDA would provide a pharmacodynamic biomarker to track the progression of enzyme inactivity in the mitochondria, the overall pathophysiology, and the response to therapy. ATP levels can be measured in muscle (skeletal and cardiac) using 31-P nuclear magnetic resonance (NMR) or analogous approaches [17,103,104]. A number of techniques have been developed to image ATP in the brain using optical microscopy paired with probes [105,106], as well as some magnetic resonance techniques for *in vivo* studies [105]. Biosensors have been used *in vivo* and in yeast [107], but none of the techniques so far are ideal, preventing them from being used in human subjects. There have also been several non-imaging approaches to quantify ATP; however, these also have failed in humans and cannot provide content equivalent to imaging methods [105]. Unfortunately, the technology has not advanced enough to make measuring ATP in the brain a safe and efficient resource in FRDA at this time.

### Metabolic function imaging

While ATP levels or synthesis cannot be imaged with current technology, metabolic labeling can examine other measurable markers such as reactants or products of the Krebs cycle. Pyruvate, lactate, and glutamate can be labeled *in vivo* by introducing heavy carbon (^13^C)-containing precursors into a subject, which can then be imaged using a 3T MRI scanner [108,109]. The ^13^C is then identified by MRI/MRS and separated into quantitative measures of the compounds into which it is metabolized, such as pyruvate, lactate, and glutamate [109]. By measuring the reactants and products of the metabolic process, overall and specific metabolic functions in the brain can be assessed in patients (such as those with FRDA) in comparison with controls. Metabolic dysfunction is a known consequence of frataxin deficiency thus potentially resulting in altered levels or flux of compounds such as GABA and glutamate [110]. Much like trying to image Krebs cycle intermediates, this technique is imperfect for a few reasons. Metabolism in the CNS involves many enzymes that are not directly tracked with this method, making it hard to draw exact interpretations of the effect of frataxin deficiency on metabolic function. This method is also more invasive (due to the need to introduce labeled substances) and expensive (the cost of the ^13^C compound) than a typical MRI [108,109].

## Conclusions

The present review has extended previous discussions on the mitochondrial pathophysiology of FRDA and how it may be used therapeutically and in future monitoring of the disorder. These events are complex, seemingly involving many different mechanisms that eventually lead to a relatively selective neurological and systemic disease—a true metabolic disease from frataxin deficiency (Figure 1). By isolating these pathways, targeting them with therapeutic strategies, and assessing them in human in clinical trials, one can logically move toward successful therapy. Further understanding of such events, and technological imaging advances to monitor mitochondrial events *in vivo* may facilitate pharmaceutical advances. However, even this approach is overly simplistic. Each step or pathway in Figure 1 does not occur in isolation; there is substantial cross-talk between the pathways. In some cases, the interactions are cooperative, leading to pathological positive feedback cycles. In other cases, they are compensatory, as nominally abnormal events block the toxicity of other pathways. In still other interactions, observations are likely to be epiphenomena, representing markers but not causes of disease. This complexity emphasizes the need to understand both the early and late events of FRDA, such that therapeutic development can become successful.

## Abbreviations

ARE: antioxidant response element
ATP: adenosine triphosphate
BSO: buthionine sulfoximine
DMF: dimethylfumarate
Fe–S: iron–sulfur
FRDA: Friedreich ataxia
GABA: γ-aminobutyric acid
GPX4: glutathione peroxidase-4
MRI: magnetic resonance imaging
MRS: magnetic resonance spectroscopy
NFK-B: nuclear factor κ B
NQO1, NADPH: quinone oxidoreductase
Nrf2: nuclear factor erythroid 2-related factor 2
PGC1α: peroxisome proliferator-activated co-activator 1-α
PPARγ: peroxisome proliferator-activated receptor-γ
QSM: quantitative susceptibility mapping
ROS: reactive oxygen species
SOD: superoxide dismutase
